# Progressive Pernicious Anæmia: A Case with Autopsy

**Published:** 1882-01

**Authors:** Edward Cranch

**Affiliations:** Erie, Pa.


### CLINICAL BUREAU.

### PROGRESSIVE PERNICIOUS ANÆMIA: A CASE, WITH AUTOPSY.

Edward Cranch, M. D., Erie, Pa.

The following is the record of a case of pernicious anæmia, or as I would also call it, a case of "Addison's Disease" without bronzing. Professor Pepper, of Philadelphia, has maintained the essential identity of these two diseases, and Addison himself makes no practical distinction between what he called "idiopathic anæmia," and the same condition accompanied by bronzing of the skin. Ziemssen classes tubercular disease of the supra-renal capsules or Addison's disease under one head, and the name in the title of this paper under another distinct head, but the two descriptions are marvellously alike, except for the mention of bronzing of the skin in one case, and of extravasations of blood in the other. In the latter case, the remarkable statement is made, that, because there is no bronzing of the skin, *therefore*, there is no disease of the supra-renal capsules, and accordingly no mention of those organs is made in describing the pathological condition. Now in the case herewith reported, there was no bronzing, but there were hemorrhages, and there was disease of the supra-renal capsules. The record is one of a partial failure, for the correct diagnosis was not made out during life; it being supposed by some that consumption was her enemy, by others, including herself, the cause of trouble was assigned to the kidneys, while I, myself, expected to find a cancer of the stomach or bowels, but was disappointed. All of these diseases, however, are mentioned as very frequent complications of pernicious anæmia, and of Addison's disease.

The patient, Miss D. P., aged 31, had commenced to complain six years before I saw her; at the first, in 1873, she had had a sore on one of her fingers, pronounced a "rose cancer," and excised without returning. Next, she was noticed to have frequent faint spells, suddenly turning pale, "as white as a corpse," afterward vomiting, headache, cough, and great lassitude. From first to last, she often complained of "terrible heat in the back, so that she would not lie up against anything," also of poor appetite, offensive urine, bland leucorrhœa, and dry cough She was not particularly nervous. When first seen by me, in August, 1879, she had a diarrhœa, offensive, watery, and containing a brownish matter like coffee-grounds, or scrapings of intestines; vomiting whenever rising up from lying down, ejecting a greenish water, with cramps in the stomach and bowels, great debility, a horribly pale, strange cast of complexion (very striking), but no emaciation, or œdema. Although it was summer, she was wrapped to the eyes in a woollen comforter, and wore woollen leggings. She got *Arsen.* CM (Fincke) six doses, twelve hours apart. This stopped the vomiting, which *never returned.* In October and November, she got *Phos.* 200, four or five times a week, for the cough, debility, heat in back, and diarrhœa, with decided improvement in everything except complexion. During the winter she was quite comfortable, attending several social parties, enjoying fair appetite, and entertaining the strongest hopes of her recovery. In April, 1880, the diarrhœa returned, the urine showed excess of urates and phosphates, but no albumen or casts, the appetite and strength failed entirely, there was some œdema of the feet, some dyspnœa, and two severe attacks of nose-bleed, requiring plugging of the posterior nares, occurred a few days before her death, which happened May 1st, 1880, quietly and without pain. A *post-mortem* examination was held May 2d, with the assistance of Dr. W. C. Evans, of Erie, an old school physician-who had never seen the case during life. On making the prelimi, nary incision we found a quantity of *adipose,* more than we expected from the extreme anæmia of the body; this at once reminded Dr. E. of a similar case he had examined, with slight bronzing, found only on thigh and scrotum. (In this case there was *none.*) The bowels were free from cancer, or other morbid product, except a slight serous effusion. The kidneys were normal in size and aspect but the supra-renal capsules were soft, yellow, very friable, and showed under the microscope a quantity of fat. They did not seem enlarged. The liver was slightly pigmented, and both liver and kidney showed an abnormal amount of fat, but nothing near the proportion that was manifest in the capsules. The heart and lungs were not examined, nor was the marrow of the bones.

This class of cases is deemed *entirely incurable*, by the old school, and *Iodine, Iron, Nitrate of Silver, Alcohol*, and transfusion of blood have been recommended. From a general view of the disease, I would study, besides *Arsenic* and *Phosphorus, Picric acid, Causticum, Carbo veg., Antimonium crudum*, and others.

